# Loss of the insulator protein CTCF during nematode evolution

**DOI:** 10.1186/1471-2199-10-84

**Published:** 2009-08-27

**Authors:** Peter Heger, Birger Marin, Einhard Schierenberg

**Affiliations:** 1 Zoological Institute, University of Cologne, Kerpener Strasse 15, 50937 Köln, Germany; 2 Botanical Institute, University of Cologne, Gyrhofstrasse 15, 50931 Köln, Germany

## Abstract

**Background:**

The zinc finger (ZF) protein CTCF (CCCTC-binding factor) is highly conserved in *Drosophila *and vertebrates where it has been shown to mediate chromatin insulation at a genomewide level. A mode of genetic regulation that involves insulators and insulator binding proteins to establish independent transcriptional units is currently not known in nematodes including *Caenorhabditis elegans*. We therefore searched in nematodes for orthologs of proteins that are involved in chromatin insulation.

**Results:**

While orthologs for other insulator proteins were absent in all 35 analysed nematode species, we find orthologs of CTCF in a subset of nematodes. As an example for these we cloned the *Trichinella spiralis *CTCF-like gene and revealed a genomic structure very similar to the *Drosophila *counterpart. To investigate the pattern of CTCF occurrence in nematodes, we performed phylogenetic analysis with the ZF protein sets of completely sequenced nematodes. We show that three ZF proteins from three basal nematodes cluster together with known CTCF proteins whereas no zinc finger protein of *C. elegans *and other derived nematodes does so.

**Conclusion:**

Our findings show that CTCF and possibly chromatin insulation are present in basal nematodes. We suggest that the insulator protein CTCF has been secondarily lost in derived nematodes like *C. elegans*. We propose a switch in the regulation of gene expression during nematode evolution, from the common vertebrate and insect type involving distantly acting regulatory elements and chromatin insulation to a so far poorly characterised mode present in more derived nematodes. Here, all or some of these components are missing. Instead operons, polycistronic transcriptional units common in derived nematodes, seemingly adopted their function.

## Background

Chromatin insulation plays a profound role in regulating gene expression and is mediated by the binding of insulator proteins to specific DNA sequence elements. So far, in only a limited number of organisms insulator function has been demonstrated: in yeast [1-3], sea urchin [4,5], *Drosophila *(e. g. [6,7]), and vertebrates (e. g. [8,9]). *D. melanogaster *and vertebrates are the only metazoan systems where insulator binding proteins have been identified. In *Drosophila*, Suppressor of Hairy Wing [Su(Hw)], Boundary Element Associated Factors (BEAF-32A and BEAF-32B), Zeste-white 5 (Zw5), GAGA Binding Factor (GAF), and, most recently, CTCF (dCTCF) have been described as functional insulator proteins. In contrast, there is only one known insulator protein in vertebrates, CTCF, which is associated with all known insulators [10,11].

CTCF was initially described as a transcriptional regulator of the chicken c-myc proto-oncogene [12]. Besides its function as a transcription factor, CTCF is important for several other cellular processes, e. g. genomic imprinting, X-chromosome inactivation, control of DNA methylation state, or long-range chromatin interactions (for review see [13]). The link between CTCF and chromatin insulation was established in 1999 with the discovery that the borders of the chicken *β*-globin locus have insulator activity and resemble CTCF binding sites necessary for insulating the *β*-globin genes and maintaining their distinct regulatory programs [14,15].

Recently, a systematic computational search for conserved noncoding elements revealed a highly enriched CTCF binding motif occurring at nearly 15.000 positions within the human genome [16]. Nearby genes separated by predicted CTCF sites exhibited a markedly reduced correlation in gene expression, consistent with the hypothesis that CTCF insulator sites partition the genome into independent domains of gene expression. A similar number of potential insulator sites was found by chromatin immunoprecipitation experiments (ChIP) against CTCF and, strikingly, the CTCF binding consensus motif deduced there was virtually identical to the enriched conserved element defined in the first study [17]. In addition, it was shown that the sites of CTCF-binding sequences in the human genome are highly conserved in other vertebrates, consistent with a widespread and fundamental role of CTCF in different organisms. More evidence of CTCF function in the establishment of discrete chromosomal domains was provided by [18] who showed that interactions between genome and nuclear lamina take place abundantly with CTCF sites preferentially demarcating the identified lamina associated domain borders. These data suggest that CTCF is an essential organiser of long-range chromatin interaction and transcription across species.

In 2005, presence of a CTCF ortholog outside the vertebrates was reported for the first time [19]. This *Drosophila *CTCF had a binding site specificity similar to vertebrate CTCF and conveyed insulator activity to one known insulator in the *Drosophila *Abdominal-B locus of the Bithorax complex. ChIP-chip experiments of the whole Bithorax complex revealed that dCTCF is directly associated with almost all known or predicted insulators in this region [20]. Binding of dCTCF to the insulators of the Bithorax complex is relevant *in vivo *because dCTCF null mutations in the fly affect expression of Abdominal-B, cause pharate lethality and a homeotic phenotype [21]. The relevance of CTCF for normal development was also illustrated in vertebrates as its elimination in mice resulted in early embryonic lethality [22].

These reports from vertebrates and flies highlight the importance of chromatin insulation and insulator binding proteins on a global genomic scale in both systems (for recent reviews, see [13,23]). Moreover, the work about dCTCF showed that a key player of chromatin insulation is conserved from fly to man. We reasoned therefore that chromatin insulation might also be a relevant mechanism of regulating gene expression in nematodes and conducted a systematic computational survey of all available nematode genomes and EST data sets to detect orthologs of the presently known insulator binding proteins.

In the following we will frequently refer to «basal» and «derived» nematodes. In a recent publication, the phylum Nematoda has been divided into 12 clades [24]. According to this classification we define members of clades 1 and 2 as «basal» nematodes, while members of clades 3 – 12 including *C. elegans *(clade 9) are designated «derived». This view of the Nematoda being divided into two major groups corresponds to their partition into the classes Enoplea (basal, paraphyletic) and Chromadorea (derived, monophyletic; see Discussion for further arguments supporting this view) [25,26].

## Results

### Known insulator binding proteins are not found in nematodes except CTCF

We searched whole genome sequence databases of seven nematode species for orthologs of the known insulator proteins Su(Hw), BEAF-32, GAGA factor, Zw5, and CTCF.

With dCTCF as query, a high scoring predicted open reading frame (ORF; e-113) was identified in the genome assembly of the basal nematode *Trichinella spiralis*, but not in the genomes of other, more derived nematodes (Table 1). Reciprocal BLAST searches of this ORF in the NCBI database confirmed a high similarity to CTCF proteins of insects and vertebrates. The same approach, when conducted with the reported hits from other nematode genomes, resulted in non-CTCF zinc finger proteins (data not shown).

**Table 1:** BLAST results for insulator protein searches in nematode genomes

	**Su(Hw)**	**BEAF-32**	**GAGA**	**Zw5**	**CTCF**
*C. elegans*	134	-	52	132	124
	125		42	129	106
Clade 9	113		42	128	100

*C. briggsae*	133	-	43	136	107
	117		42	128	105
Clade 9	114		39	125	101

*C. remanei*	134	-	53	129	131
	127		47	128	107
Clade 9	119		47	125	103

*P. pacificus*	69	32	31	73	60
	69	32	31	73	59
Clade 9	63	-	30	68	56

*A. suum*	75	-	40	76	56
	67		35	71	55
Clade 8	63		34	62	49

*B. malayi*	178	-	37	197	192
	174		37	197	181
Clade 8	173		36	196	181

*T. spiralis*	134	30	45	146	**408**
	116	29	40	134	141
Clade 2	105	-	33	105	107

Results of BLAST searches for five known insulator proteins in seven nematode genomes (clade numbering after [24], see Figure 4A). Numbers represent the reported BLASTP score of the three best hits in a given genome. Only CTCF can be detected in the basal nematode *T. spiralis *(bold). See text for further explanation.

When we searched for Su(Hw) or Zw5 orthologs, relatively high BLAST scores were generated in some nematode genomes (Table 1). Reciprocal BLAST analysis at the NCBI website however showed that, in all cases, these could be attributed to a number of adjacent C2H2 zinc finger domains and never traced back to an insulator protein query.

The insulator proteins BEAF-32A and B, which do not contain ZFs, and GAGA factor, having a single ZF, did not produce significant hits in our nematode data set, suggesting the general absence of a related protein in nematodes (Table 1).

Searches for Su(Hw), Zw5, and CTCF orthologs in *Brugia malayi *resulted in considerably higher scores compared to other nematode genomes (Table 1). But again, reciprocal best BLAST could not unveil a link to known insulator proteins (data not shown). Remarkably, however, these scores are produced from a family of ZF proteins consisting of at least 15 extraordinary similar members with multiple adjacent ZFs whose most similar sequences in humans are the KRAB containing ZF proteins 235 and 93 (Q14590 and NP_004225). A similar family of nearly identical ZF proteins is absent in other nematode genomes (PH, unpublished data).

In contrast to the *Caenorhabditis *species, an annotated set of the protein coding regions is not yet available for the *Ascaris suum *and *Pristionchus pacificus *genomes, restricting our data set to the possible ORFs derived from the preliminary sequence assembly. Therefore, BLAST analysis can reveal only single similar ORFs and not whole annotated proteins, explaining the lower average scores in these organisms (Table 1).

We noticed that BLAST analysis with the ZF proteins Su(Hw), Zw5, and CTCF generated the following partially overlapping gene matches in the *C. elegans *genome: F25D7.3; Y55F3AM.14; Y38H8A.5; C55B7.12; F45B8.4; R12E2.1; R11E3.6; T27E9.4 (for information see [27]). For three of these proteins, the function is known, e. g. as a transcription factor in specific cells [28-30]. For the remaining proteins no functional data or mutant alleles exist and their RNAi-mediated knockdown did not result in observable phenotypes (except in F25D7.3) [27]. Therefore, the available information about the above mentioned *C. elegans *proteins gives no indication that they might act as insulator proteins [27]. The same conclusions also apply to the *C. briggsae *and *C. remanei *genomes.

To extend our data set, we conducted BLAST searches with the same five insulator protein sequences in all available nematode ESTs ([31]). Consistent with our previous results (Table 1), we could not obtain candidates for any of the known insulator proteins after reciprocal BLAST tests (data not shown) except for CTCF. We identified CTCF orthologs in two out of three other basal nematodes, in *Xiphinema index *(clone XI00686, 5.9e-73) and *Trichuris muris *(clone TM01708, 1.6e-62), but not in the ESTs of 32 derived nematode species.

Taken together, our results suggest that the whole nematode phylum apparently does not possess known insulator proteins, except orthologs of the genome organiser and insulator protein CTCF which seems to be restricted to basal nematodes.

### Cloning and characterisation of CTCF from the basal nematode T. spiralis

**Figure 1:**
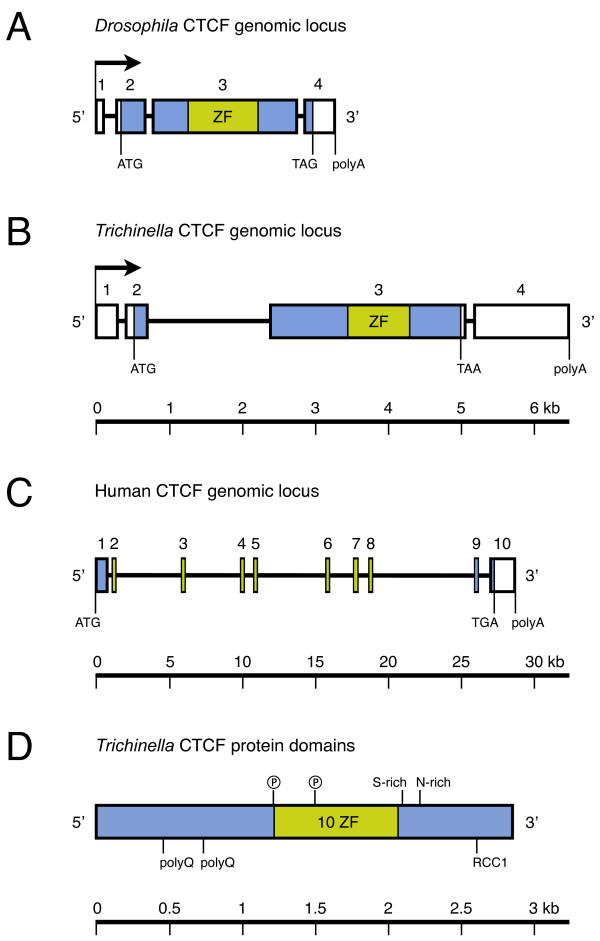
**Conserved genomic organisation of invertebrate CTCFs**. **A, B, C**: Genomic CTCF loci of vertebrates, *Drosophila *[19] and *Trichinella*, drawn to scale. Transcription start (arrow), transcription end (polyA), translation start (ATG) and stop (TAA, TAG, TGA), exons (box), introns (line), protein coding region (shaded), and zinc finger region (light green) are indicated. To illustrate the structure of vertebrate CTCFs, human CTCF is depicted (accession number NT_010498). **D**: Domain organisation of tsCTCF. Ⓟ: Predicted Tyrosine phosphorylation site, polyQ: poly-Glutamine tract, S-rich: Serine-rich region, N-rich: Asparagine-rich region, RCC1: RCC1 chromatin binding motif, ZF: Zinc finger region. Predicted N-glycosylation, N-myristoylation and most phosphorylation sites are not shown.

To confirm our computational identification of putative CTCF orthologs in basal nematodes, we cloned the mRNA of the *T. spiralis *CTCF ortholog (tsCTCF). Comparison with the unpublished *T. spiralis *genome sequence assembly (accession number ABIR01000000) revealed that tsCTCF lies on a 6.5 kb genomic locus. The primary transcript contains four exons, with the first and last being untranslated, and three introns, with a large second intron (1.7 kb). The resulting 4.6 kb mRNA has a 414 bp 5'UTR and a 1328 bp 3'UTR, both harboring a small intron of about 100 bp. The deduced protein coding region (948 AA) is a fusion of two exons, a small first one and a large second exon carrying the entire ZF region (Figure 1B).

A comparison of the genomic structure reveals remarkable similarities between *Trichinella *and *Drosophila *CTCF, the most closely related published CTCF ortholog (Figure 1A, B). Both invertebrate CTCFs contain only four exons while ten small exons are scattered over a large genomic locus in all vertebrate CTCFs (Figure 1C). The entire ZF region of the invertebrate CTCFs is located on a large exon comprising ≥80% of the coding region while it is composed of seven short exons in vertebrates [32]. The 5'UTR, interrupted by an intron, is leading in frame to the translation start in both, *Trichinella *and *Drosophila *CTCF. However, unlike dCTCF the *Trichinella *gene carries a larger 3'UTR (1328 bp versus 320 bp) that is also present in vertebrate CTCFs (1413 bp for the chicken CTCF 3'UTR [33]).

**Figure 2:**
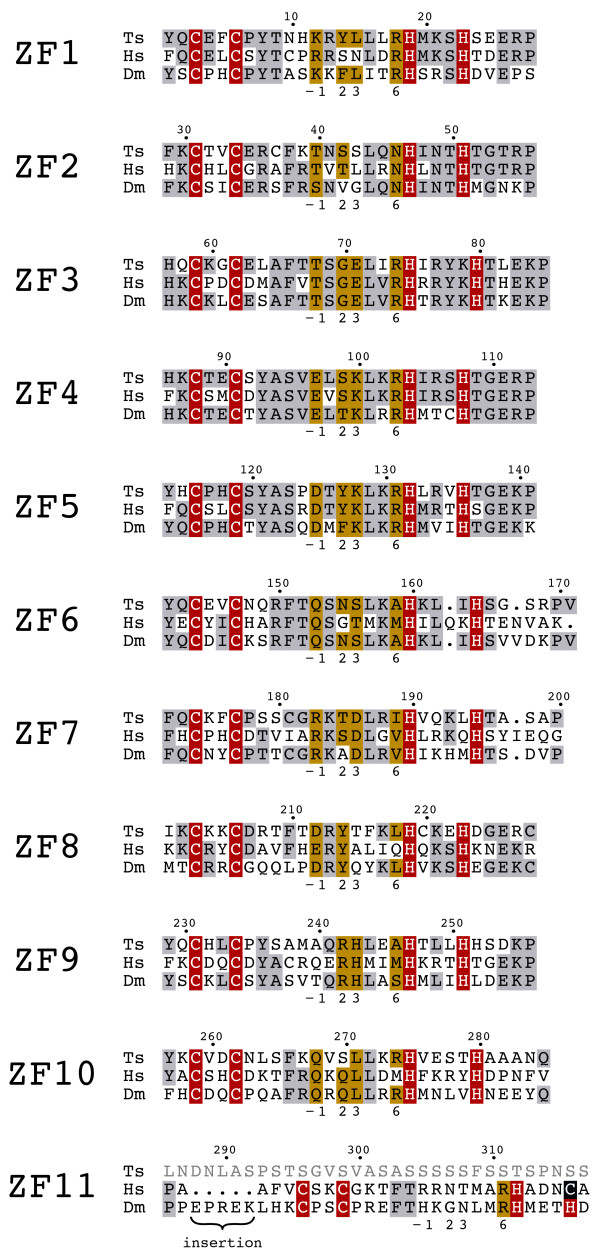
**Conserved zinc finger region of tsCTCF**. Multiple sequence alignment of the ZF domains of *T. spiralis*, human, and *D. melanogaster *CTCF. Similar and identical residues (grey), zinc coordinating residues (red, blue), and identical residues for DNA recognition (yellow) are indicated. ZF11 is missing in *T. spiralis *and is less conserved between *D. melanogaster *and man than ZF1 – 10.

The central region of the protein contains ten C2H2 ZFs conserved in all reported CTCF sequences [19,32]. Within vertebrates, the eleventh ZF is of the C2HC-type. In *Drosophila *however, ZF11 is a C2H2-type finger and displays only weak conservation of the critical DNA binding residues as well as a small insertion (Figure 2). In tsCTCF, ZF11 is missing entirely, and neither 3'Race PCR nor the genomic sequence at this locus gave indications for its presence. These observations suggest that conservation of ZF11 is not a strict requirement for functional CTCF proteins.

Figure 2 depicts an alignment of the ZF region of human, *Trichinella*, and *Drosophila *CTCF. Most of the crucial DNA recognition residues at positions -1, 2, 3, and 6 are identical between at least two of the three species. Variations in position 6 for ZF6 and ZF9 generate a change from alanine or serine to methionine, which does not alter the DNA recognition code of the finger [19,34]. The identity within the ZF region between the human and *Trichinella *proteins is 52%, exceeding the rate of 44% between *Drosophila *and vertebrates (Table 2). Flies, however, belong to a highly derived insect order with rapid evolution [35,36]. We therefore extended our analysis to CTCF sequences from more «basal» insects, *Apis mellifera *and *Tribolium castaneum*. The CTCFs from both, basal nematodes and basal insects, are more similar to the human counterpart and to each other than *Drosophila *CTCF (Table 2). The lower similarity of *Drosophila *CTCF is therefore probably due to the rapid evolution of the fly insect order.

**Table 2:** Similarity of CTCF zinc finger regions

	Identity (%)	Similarity (%)
Dm/Hs	44	59
Am/Hs	53	68
Ts/Hs	52	68

Ts/Dm	60	77
Ts/Am	70	84
Dm/Am	67	80

Percent similarity and identity of the *D. melanogaster*, *A. mellifera*, *T. spiralis*, and human CTCF ZF regions. The identity between human CTCF (Hs) and CTCF from basal nematodes and basal insects (Ts, Am) is higher than between human CTCF and *D. melanogaster *CTCF, a rapidly evolving insect (Dm). Values result from the comparison of human (Accession number NM_006565.2), *A. mellifera *(XP_624731.1), *D. melanogaster *(NP_648109.1), and our *Trichinella *CTCF.

CTCFs contain conserved motifs in the N- and C-terminal domains which are important mainly for protein-protein interaction [37]. Prominent candidates for such motifs could not be found in the *Trichinella *N- and C-terminal domains. In particular, an AT hook motif described for *Drosophila *and vertebrate CTCFs is absent in *Trichinella*. Nevertheless, scanning the coding region in the PRINTS [38] and PROSITE databases [39] generated several matches. Consistent with the presumed DNA binding function of the protein, a putative RCC1 chromatin binding motif was detected near the C-terminus (AA 869 – 886) that is not present in other known CTCFs. Commonly found post-translational modifications like N-glycosylation (6 instances), N-myristoylation (14 instances), and phos-phorylation (22 instances, including cAMP-dependent kinase, Casein kinase II, Protein kinase C, and Tyrosine kinase sites) were also predicted for tsCTCF, the latter being consistent with the presence of functional phosphorylation sites in vertebrate CTCF [40]. In addition, pronounced Glutamine-rich regions (AA 148 – 168 and 252 – 338), a Serine-rich region (AA 698 – 723), and an Asparagine-rich region (AA 736 – 766) are present in *Trichinella*, but not in other CTCFs (Figure 1D).

Despite minor differences, the similar genomic organisation of *Trichinella *CTCF and *Drosophila *CTCF as well as the remarkable conservation of the ZF domain and DNA binding residues point to a conserved function of this protein in the basal nematode *Trichinella spiralis*. This assumption is further supported by our work in progress that demonstrates a specific binding of *Trichinella *CTCF to known *Drosophila *CTCF target sites in electric mobility shift experiments, indicating that the binding preferences of the two proteins are very similar (M. Bartkuhn and P. Heger, unpublished data).

### Loss of CTCF during nematode evolution

In our initial survey we identified several putative CTCF orthologs in basal nematodes while in derived nematodes like *C. elegans *or *B. malayi *a protein with a high similarity to CTCF could not be detected. This raises the possibility that originally CTCF was present in nematodes, but was lost during nematode evolution.

**Figure 3:**
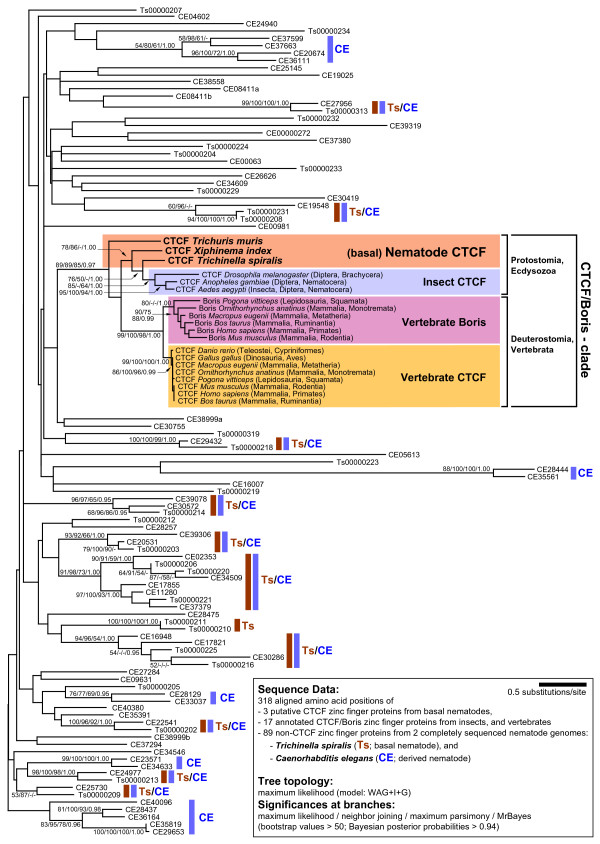
**Presence of CTCF in basal nematodes**. Phylogenetic analysis of ZF proteins from nematodes, insects, and vertebrates. The tree topology resulted from protein maximum likelihood, using the model WAG+I+G. To test the robustness of the branching pattern, three bootstrap analyses were performed with protein maximum likelihood (WAG+I+G), neighbor joining, and maximum parsimony methods. Bootstrap support values >50% are shown. Bayesian posterior probabilities, resulting from a Bayesian analysis (model: WAG+I+G), served as additional measure for the confidence of branches. Posterior probabilities >0.94 were considered as significant, lower values were ignored. Ts000x: sequence from *T. spiralis*, CEx: sequence from *C. elegans*. All putative as well as annotated CTCF and Boris orthologs form a single, well-supported clade. Note the paralogous clusters of vertebrate CTCF and vertebrate Boris [32] within this clade. No ZF protein of the derived nematode *C. elegans *clusters to the known CTCFs, whereas a single ZF protein of *T. spiralis *and CTCFs of two other basal nematodes do so. Several significantly supported clades of non-CTCF zinc finger proteins contain orthologs of both nematode genomes analysed here (Ts/CE), indicating an early diversification of ZF proteins.

To test this hypothesis we performed a phylogenetic analysis with a C2H2 ZF protein alignment that contained (i) three putative CTCF orthologs from basal nematodes (*Trichuris muris*, *Xiphinema index*, and *Trichinella spiralis*), (ii) 17 annotated CTCF proteins from insects and vertebrates (including «Boris» sequences, a vertebrate CTCF paralog), and (iii) 89 selected C2H2 ZF proteins (see Methods) derived from the *Trichinella spiralis *and *Caenorhabditis elegans *genome sequences (Figure 3).

Irrespective of the method of tree reconstruction, our phylogenetic analysis recovered all known and putative CTCF orthologs as a well-supported gene family, the CTCF/Boris-clade (Figure 3). A single sequence of the basal nematode *T. spiralis *clustered to the CTCF/Boris-clade, whereas all remaining ZF proteins of the *Trichinella *genome formed many independent branches/gene families, separate from the CTCFs. Putative CTCF sequences from two other basal nematodes, *Trichuris muris *and *Xiphinema index*, were also resolved as members of the CTCF/Boris-clade. The phylogenetic position derived for the three nematode CTCFs is nearest to insect CTCFs, emphasising the close genomic similarity observed between *Trichinella *and *Drosophila *CTCF. These data confirm that our isolated *Trichinella *protein is indeed a member of a unique group of CTCF proteins in invertebrates.

In contrast, ZF proteins from the derived nematode and standard model system *Caenorhabditis elegans *behave differently. Here, all ZF proteins are clearly apart from the CTCF/Boris-clade, indicating that a CTCF ortholog is not present in *C. elegans *(Figure 3). We wanted to confirm these findings with corresponding ZF protein data sets from two closely related *Caenorhabditis *species, *C. briggsae *and *C. remanei*, and, in both cases, no ZF protein clustered to the CTCF/Boris-clade (data not shown).

When we included in our analysis a ZF set derived from the *Drosophila melanogaster *genome, only the single known CTCF ortholog of this species joined the CTCF/Boris-clade as in Figure 3 (data not shown).

Non-CTCF ZF proteins of the nematodes *Trichinella *and *Caenorhabditis *displayed high sequence diversity, and thus, our phylogenetic analysis largely failed to resolve the ancestral diversification of non-CTCF ZF proteins. Nevertheless, it recovered many significantly supported terminal clades (gene families) with two to several members, respectively (Figure 3). Most non-CTCF gene families are present in both, *Trichinella *and *Caenorhabditis *(«Ts/CE» in Figure 3), whereas some others are confined to only one genome («Ts» or «CE» in Figure 3). These results suggest an early diversification of ZF protein families, which significantly predated the split between basal and derived nematode lineages.

Derived (e. g. *Caenorhabditis*) and basal nematode clades (e. g. *Trichinella*) are separated by about 700 million years of evolution [41,42]. To more precisely determine when CTCF was lost during nematode evolution, we included ZF sets from nematodes positioned between *Trichinella *and *Caenorhabditis*. However, we did not find CTCF-like proteins in *B. malayi *and *A. suum *(clade 8; data not shown), which are separated from *C. elegans *by an estimated 350 million years [43].

Inclusion of additional CTCF-like proteins from various invertebrates confirmed the position of the nematode CTCFs within the CTCF/Boris-clade, close to insect CTCFs (data not shown).

Taken together, our phylogenetic results indicate that a CTCF ortholog is present in nematodes, but only in their most basal clades.

## Discussion

The fundamental role of chromatin insulation in the regulation of gene expression is increasingly being recognised in vertebrates and fly [16,20,44,45]. However, whether the underlying mechanisms and proteins are conserved throughout the animal kingdom is not known. Therefore, we conducted a first systematic approach to detect orthologs of known insulator proteins in a phylum more primitive than vertebrates and insects, in nematodes. While we could not find orthologs of other known insulator proteins, we detected orthologs of CTCF in basal, but not in derived nematodes.

As two of these basal nematodes, *Trichinella spiralis *and *Trichuris muris*, are vertebrate parasites, a simple explanation for the presence of CTCF could be horizontal gene transfer (HGT) from host to parasite.

However, our phylogenetic analysis clearly rejects this possibility (Figure 3). The nematode CTCFs do not cluster to the vertebrate CTCF proteins as one would expect for a HGT scenario. Instead, they are positioned at the root of the fly CTCF cluster. Implementation of additional CTCF-like sequences from other arthropods reinforces this finding (data not shown). Furthermore, we show that *Xiphinema index*, a basal plant parasitic nematode, contains CTCF. But BLAST searches indicated that a CTCF-like protein is not present in available plant genome sequences including *Arabidopsis thaliana *and grapevine (*Vitis vinifera*), a common host of *Xiphinema index *(not shown).

Therefore, we assume that CTCF was originally present in nematodes. Our finding of a CTCF ortholog only in basal nematodes allows several possible scenarios for the evolution of gene expression in the phylum Nematoda. One possibility is that the original function of CTCF is not related to chromatin insulation, but that insulation properties appeared later in evolution. As both, fly and vertebrate CTCF, are insulator proteins, this event then must have happened independently twice or CTCF must have lost insulator activity in nematodes. To ultimately answer this question functional data for nematode CTCFs are required. However, our work in progress argues for a functional conservation of CTCF in nematodes as the DNA binding properties of *Trichinella *CTCF are very similar to *Drosophila *CTCF (M. Bartkuhn and P. Heger, unpublished data).

In a second scenario the identified protein is a true CTCF ortholog with insulator and genome organiser activity like the vertebrate and *Drosophila *counterparts. If CTCF performs these essential functions in basal nematodes, how can we explain its loss in derived nematodes like *C. elegans*? It is conceivable that during nematode evolution CTCF was not lost, but has been altered to an extent that prevents its recognition, especially as *C. elegans *and other members of the nematode crown clades 8 – 12 are fast evolving organisms [24]. As the great majority of the analysed nematode genome and EST data belongs to fast evolving species, we cannot rule out this scenario. Nevertheless, explaining the absence of CTCF with a single event like a gene loss or chromosomal deletion early in nematode evolution appears more likely than independent evolutionary loss of CTCF in so many derived nematodes.

There are CTCF-dependent functions beyond chromatin insulation which are as much as important for the viability of an organism, e. g. X-chromosome inactivation, DNA methylation, or genomic imprinting. However, several arguments support the conclusion that the loss of a central player in these functions is not deleterious for *C. elegans*. Instead of random X-chromosome inactivation involving CTCF, like in mammals, *C. elegans *uses an alternative dosage compensation mechanism to repress X-linked genes which is studied in detail (for review see [46]). DNA methylation and genomic imprinting are unknown in *C. elegans *and other derived nematodes [47-50]. A transcription factor activity of CTCF could have been passed on to other proteins. Therefore, also these additional functions of CTCF are compatible with the absence of CTCF in derived nematodes.

If CTCF and therefore CTCF-mediated chromatin insulation are absent in those nematodes, could other proteins have acquired insulator function? Presently, no data are available to support or reject this hypothesis as chromatin insulation in *C. elegans *or other nematodes is unknown, so far. However, it has been suggested that generally gene expression in *C. elegans *is controlled by regulatory elements located immediately upstream of the transcription unit [51-53]. This seems to be different to *Drosophila *and vertebrates where long-range interactions with distant regulatory elements over more than 10 kb have been reported [54,55].

Thus, our finding of the insulator protein CTCF in basal nematodes and the available data from *C. elegans *open the possibility that chromatin insulation is absent in derived, but present in basal nematodes. This would be in line with several other reports that underscore substantial differences between basal nematodes and the derived model organism *C. elegans*. Here, four examples are given. (i) Embryogenesis of *C. elegans *and other derived nematodes is characterised by a unique type of gastrulation not found elsewhere in the animal kingdom while in the basal nematode *Tobrilus diversipapillatus *(clade 1) gastrulation resembles the «classical» pattern found all over the animal kingdom [56]. (ii) Hedgehog and Smoothened, parts of the Hedgehog signaling pathway, are not present in *C. elegans *and other derived nematodes [57], but recent studies identify a *bonafide hedgehog *gene in the basal nematodes *Trichinella spiralis *and *Xiphinema index *[58]. (iii) *C. elegans *contains a greatly reduced Hox gene complement [59] while in the basal nematode *T. spiralis *several additional Hox genes were identified, suggesting Hox gene loss during nematode evolution [60]. (iv) Embryogenesis of *Romanomermis culicivorax*, like *Trichinella *a representative of the basal nematode clade 2, has revealed several fundamental differences to *C. elegans*, for example with respect to cell division patterns and tissue formation [61,62].

Looking at these prominent differences, it appears not unlikely that a fundamental difference in the regulation of gene expression exists between basal and derived nematodes. In support of this view an additional argument can be made that directly points toward such a difference in genome organisation.

*C. elegans *has operons, clusters of closely spaced genes under the control of a single regulatory signal. A genomewide survey revealed more than 1.000 operons in the *C. elegans *genome comprising about 15% of all genes [63]. Therefore, operons have to be considered a major mode of transcriptional regulation. Although structurally different, operons are functionally similar to chromatin domains demarcated by insulator proteins as both ensure coordinated expression of enclosed genes, independently from other transcriptional units.

**Figure 4:**
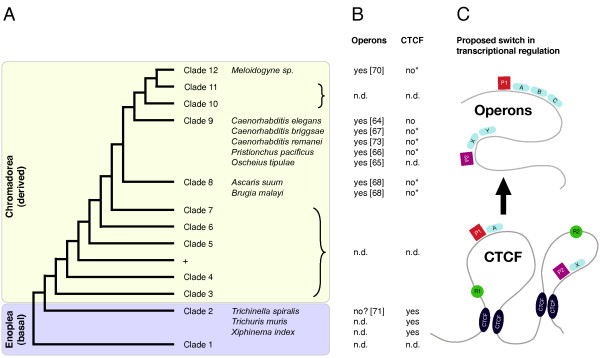
**Phylogeny and operon structure in nematodes**. **A**: Phylogenetic tree of the phylum Nematoda after [24]. Species mentioned in this study are indicated and mapped to their respective clade. A split between basal (Enoplea) and derived (Chromadorea) nematodes is well supported (see [25,26] and Discussion) and highlighted by color. **B, C**: Proposed model for a reorganisation of gene expression during nematode evolution. The presence of operons (references in parentheses) and CTCF (this study) is mapped onto the nematode phylogeny. Their mutually exclusive correlation suggests a switch during nematode evolution from transcriptional units defined by CTCF to those defined by operons (see discussion for details). With increasing formation of operons, CTCF and distant regulatory elements became obsolete. *: our data from BLAST and HMMer searches in the corresponding genome sequence. n. d.: no data. P1, P2: promoter. R1, R2: regulatory element (e. g. enhancer, silencer). A, B, C, X, Y: genes.

Operons have been shown to exist not only in *C. elegans *[64], but also in several distantly related nematodes [65-70]. However, these species all belong to the more derived nematode clades 8 – 12 (Figure 4B). Whether basal nematodes like *T. spiralis *also have their genome arranged in operons, remains to be determined. However, analysis of spliced leader trans-splicing gave no evidence that operons exist in *T. spiralis *[71].

Based on our findings and supported by other major differences between basal and derived nematodes, we propose the following model (Figure 4): In ancestral nematodes, genome organisation and transcriptional regulation were similar to the situation in *Drosophila *and operons were not present like in the great majority of eukaryotes. To ascertain coordinated gene expression, CTCF-mediated chromatin insulation was used. Trans-splicing already existed in these ancient nematodes [71,72]. Therefore, the availability of a trans-splicing machinery allowed formation of operons by providing a mechanism to express their downstream genes. As operon gains outbalance operon losses [73], they eventually became a major mode of transcriptional organisation in nematodes, superseding chromatin insulation. With decreasing selection pressure, CTCF, the mediator of chromatin insulation, finally got lost. As all analysed representatives of clades 8 – 12 have operons (Figure 4B), but presumably not *Trichinella*, a clade 2 nematode, we place that event to the split between basal (Enoplea) and derived (Chromadorea) nematodes (Figure 4C).

## Conclusion

Chromatin insulation is a fundamental feature of transcriptional regulation in eukaryotic genomes. Despite its importance, it is not known so far, whether chromatin insulation also exists in nematodes. By identifying CTCF, a mediator of chromatin insulation in vertebrates and *Drosophila*, our study reports for the first time that chromatin insulation might also be used for gene regulation in nematodes. We show that CTCF is restricted to the most basal nematode clades. From the absence of CTCF in derived nematodes we conclude that alternative gene regulation mechanisms developed early in nematode history allowing the loss of CTCF and possibly CTCF-mediated chromatin insulation. Attractive candidates for such a mechanism are operons, multicistronic transcription units that appear to be present in all derived nematodes. The striking correlation between presence of operons and absence of CTCF in nematodes suggests that operons replaced traditional transcription units based on chromatin insulation and CTCF.

## Methods

### Sequence database construction

For searching insulator proteins in *C. elegans*, Wormpep version 165 was downloaded from [27]. For analysis of the *B. malayi *and *P. pacificus *genomes, the respective published whole genome sequence assemblies [69,74] were downloaded and translated into the six ORFs using Emboss [75] omitting very short ORFs of less than 28 amino acids, the approximate size of a ZF. The same procedure was applied to the *C. elegans *genomic sequence, to exclude the possibility of a protein missing in the annotated Wormpep data set, to the unpublished *T. spiralis *whole genome assembly version 1.0, and to the unpublished *A. suum *whole genome assembly. For control purposes, the genomes of *C. briggsae *[67] and *C. remanei *(unpublished) and the proteome set of the fly *Drosophila melanogaster *(downloaded from NCBI) were included.

To detect possible CTCF orthologs, the sequence sets were scanned directly with multiple CTCF tailored HMM profiles (see below). In addition, standard BLASTP searches were conducted with known insulator proteins as queries after constructing BLAST databases from the ORF sequence sets using the BLAST suite [76]. Based on these results, we included at least the best scoring 50% of the C2H2 ZF repertoire of a genome in our data set.

### Generation of HMM profiles

A recent publication suggested that only four of CTCF's 11 ZFs are essential for strong binding [77]. Therefore we considered ZFs 4 – 7 an adequate marker for this protein and constructed an HMM profile of this region with sequences from eight organisms as input (five arthropods, two nematodes, one mammal). The profile was used to scan the available nematode genomes for matching ZF proteins. In addition, a second profile representing a single ZF motif was constructed from CTCF ZFs 4 – 8 of the same organisms. The hits obtained with both profiles were included into the data set for phylogenetic analyses. As a threshold, a HMMer score of ≤1 was defined to include virtually all multiple C2H2 ZF proteins of the respective organsim. For HMM profile generation and genomic scans the HMMER software was employed (, [78]).

### Multiple sequence alignment

BLAST and HMMer hits were combined into a non-redundant set of ZF sequences for each organism. Initial tests showed that a meaningful alignment was not possible using the raw data set due to the heterogeneity of the included proteins. We therefore restricted the data set to the ZF regions. If a protein had two or more contiguous ZF domains separated from each other, only the domain with the higher BLASTP and HMMer score was retained. Sequences with less than three ZFs were excluded from analysis. Proteins with more than 11 ZFs were trimmed to retain the 10 – 12 most similar ZFs. Although the known CTCFs have 11 ZFs, proteins with three to thirteen ZFs were ultimately allowed in the analysis. Multiple sequence alignment of the resulting data was performed using the Muscle program [79]. Alignments were viewed and edited using SeaView and TEXshade [80,81].

### Phylogenetic Analyses

Our phylogenetic data sets contained sequences representing virtually all multiple C2H2 ZF proteins of the respective nematodes. As a positive control, eight vertebrate CTCF and six Boris (Brother of Regulator of Imprinted Sites, a CTCF paralog in vertebrates) sequences were included that formed distinct clusters in a previous study [32]. In addition, three published CTCF sequences from insects were incorporated [19,82]. An outgroup was not defined.

Phylogenetic trees resulting from the alignments were computed using four different methods of tree reconstruction: maximum likelihood, neighbor joining, maximum parsimony, and a Bayesian analysis. At first, the optimal model of sequence evolution was determined by ProtTest version 1.4 [83] according to the Akaike Information Criterion. The resulting optimal model (WAG+I+G) was used for maximum likelihood (with 100 bootstrap replicates) as well as Bayesian analyses with the programs PhyML version 3.0 [84] and MrBayes version 3.1.2 [85]. For Bayesian analyses, two MCMC chains with 500.000 generations were performed, and the first 100.000 generations discarded as «burnin». To determine Bayesian posterior probabilities, a 90% majority-rule consensus of the remaining 400.000 generations was calculated. Neighbor joining and maximum parsimony bootstrap analyses (each with 100 bootstrap replicates) were performed with the program PAUP version 4.0b10 [86]. The likelihood tree was initially visualized with TreeViewPPC version 1.6.6 [87], and then graphically edited with Adobe Illustrator software.

### Cloning of tsCTCF

A BLAST database containing the ORFs of the unpublished *Trichinella *genome assembly was constructed. Herein, BLASTP searches with known CTCF queries revealed a remarkably similar ORF in Contig10.39 (e-89 for homo/mouse, e-113 for *Drosophila*). To clone this putative CTCF, RNA from adult *T. spiralis *(kindly provided by David Guiliano, Imperial College, London, UK) was used for cDNA synthesis followed by a modified Smart Race PCR protocol (Clontech Laboratories, Inc.). 3' Race PCR with a gene specific primer (ccgaagggtaactgcgagtcgatgg) resulted in a 3.1 kb fragment. To clone the 5' end, PCRs were performed with a common reverse primer and a set of forward primers situated upstream of the cloned 3' fragment in *Trichinella *Contig10.39 and spaced 200 – 250 bp apart from each other. The largest amplified fragment was sequenced, and this information was used to obtain the full length cDNA of tsCTCF via PCR. With primers (ataagatctatgcagcatgacacggccac) and (atactcgagacaaggaccggaccaaccgac) the entire coding sequence of tsCTCF was amplified from cDNA, resequenced and verified to be correct. Using sequence information from the unpublished *T. spiralis *genome we cloned and resequenced also the genomic DNA corresponding to our tsCTCF mRNA and found 100% agreement with the unpublished genome sequence. Amplification products were cloned into pJet1 vector (Fermentas). For plasmid preparation, XL1-Blue bacteria (Stratagene) were grown at room temperature (25°C) to prevent plasmid loss. For sequence annotation, the Artemis program was employed [88]. Sequence assembly was performed with the Phred/Phrap/Consed package [89,90]. Primers were designed using the Primer3 program [91]. The sequences of the *Trichinella *CTCF genomic locus and the corresponding mRNA were deposited in the EMBL Nucleotide Sequence Database (accession numbers FM991920 and FM991921).

## Authors' contributions

PH conceived the study, cloned the gene, generated the sequence databases and sets for phylogenetic analyses and wrote the manuscript except phylogeny. BM carried out and described the phylogenetic analyses. ES participated in design and coordination of the study and critically revised the manuscript. All authors read and approved the final manuscript.
